# Endothelialization of TiO_2_ Nanorods Coated with Ultrathin Amorphous Carbon Films

**DOI:** 10.1186/s11671-016-1358-0

**Published:** 2016-03-15

**Authors:** Hongpeng Chen, Nan Tang, Min Chen, Dihu Chen

**Affiliations:** State Key Laboratory of Optoelectronic Materials and Technologies, Sun Yat-sen University, Guangzhou, 510275 People’s Republic of China; School of Pharmacy, Guangdong Medical University, Dongguan, 523808 People’s Republic of China

**Keywords:** Cytocompatibility, Hemocompatibility, TiO_2_ nanorods, Amorphous carbon coatings, Nanocomposites, Human umbilical vein endothelial cells

## Abstract

Carbon plasma nanocoatings with controlled fraction of sp^3^-C bonding were deposited on TiO_2_ nanorod arrays (TNAs) by DC magnetic-filtered cathodic vacuum arc deposition (FCVAD). The cytocompatibility of TNA/carbon nanocomposites was systematically investigated. Human umbilical vein endothelial cells (HUVECs) were cultured on the nanocomposites for 4, 24, and 72 h in vitro. It was found that plasma-treated TNAs exhibited excellent cell viability as compared to the untreated. Importantly, our results show that cellular responses positively correlate with the sp^3^-C content. The cells cultured on high sp^3^-C-contented substrates exhibit better attachment, shape configuration, and proliferation. These findings indicate that the nanocomposites with high sp^3^-C content possessed superior cytocompatibility. Notably, the nanocomposites drastically reduced platelet adhesion and activation in our previous studies. Taken together, these findings suggest the TNA/carbon scaffold may serve as a guide for the design of multi-functionality devices that promotes endothelialization and improves hemocompatibility.

## Background

Anti-thrombogenicity and endothelialization are two essential issues in devising blood-contacting medical implants, such as artificial blood vessels and vascular stents [1, 2]. Minimizing the plasma protein adsorption and platelet adhesion has proved beneficial in reducing thrombus formation especially in the initial implantation. Subsequently, rapid endothelialization of implant surfaces may significantly reduce the risk of long-term thrombogenesis and provide a fully hemocompatible interface. Furthermore, native endothelium has unique physiological role of maintaining vascular homeostasis, including the active anti-thrombosis, and the release of soluble factors that contribute to the inhibition of smooth muscle cell proliferation and hence reduce intimal hyperplasia [3, 4]. Rapid regeneration of endothelium is thereby crucial to the success of implantation. Numerous approaches such as natural polymer coating (collagen) [5], surface biomolecule immobilization (heparin) [6], and drug-eluting coatings (paclitaxel) [7] have been demonstrated to be able to decrease the risk of thrombosis, but the instability, temporality, and the side effect limit their clinical use.

The nano- and microstructure of surfaces with physical attributes has been established as a decisive factor affecting biological responses. Sub-micrometer textures [8], poly(carbonate urethane)-coated carbon nanotube [9], TiO_2_ nanotube layers [10], and lotus-leaf-like structured polymer film [11], have been reported to remarkably decrease the activation and adhesion of platelets. However, these surfaces exhibited superhydrophobicity (CA > 150°) or approximately superhydrophobicity and focus on hindering only the adhesion of platelets to surfaces. In most cases, cell function was found be suppressed on the highly hydrophobic materials [12, 13]. Hence, ideal blood-contact biomaterials should maintain good anti-thrombogenicity and has positive effects on cell behavior. Recently, Ding et al. suggest that the anisotropic pattern featuring 1-μm grooves could enhance endothelialization and reduce platelet adhesion and activation [14].

Amorphous and crystalline carbon films deposited on metals have been studied as possible candidates for biomedical applications, mainly because of their chemical inertness, lack of cytotoxicity, and the natural presence of this element in the human body [15, 16]. The TiO_2_ nanorod arrays (TNAs) showed outstanding blood compatibility due to its special surface topography and hydrophobicity in our previous work [17]. After being coated with a-C films, the hemocompatibility of TNA nanocomposites was better than that of the separate TNA or a-C films [18, 19]. In this work, we reported the in vitro bioactivity and cytocompatibility of human umbilical vein endothelial cells (HUVECs) on the TNA/carbon films with different sp^3^-C bonding but independent of surface topography. Our data clearly demonstrate that amorphous carbon films significantly improve cell viability and suggest the possibility that sp^3^-C containing in carbon films must have been one of the important factors contributing to the wettability and cell cytocompatibility of TNA/carbon nanocomposites.

## Methods

### Synthesis of TNA/ta-C Composites

The single crystal rutile TNAs was synthesized on a piece of fluorine-doped tin oxide (FTO) glass substrates by the solvent-thermal method. 22.5-mL deionized water was mixed with 17.5-mL hydrochloric acid (36.5–38 % by weight) to reach a total volume of 40 mL in a Teflon-lined stainless steel autoclave (100 mL). The mixture was stirred for 5 min before the addition of 0.4 ml tetrabutyl titanate (99 % J&K Scientific). After stirring for another 5 min, four pieces of FTO substrates (3.0 × 0.5 × 0.2 cm^3^), separately cleaned with sonication in acetone, ethanol, and deionized water each for 15 min, were leaned against the wall of the Teflon-liner with the conductive side facing down. The solvent-thermal synthesis was conducted at 140 °C for 6 h. After the synthesis, the autoclave was cooled to room temperature and the FTO substrate was taken out, rinsed with deionized water, and dried in nitrogen stream. Subsequently, the oriented TNAs grown on FTO substrates were subjected to pure C^+^ ion flux produced by the FCVAD system. C^+^ plasma was generated by igniting an electric arc between a mechanical trigger and a graphite cathode (99.99 %) with a continuous DC current of 50 A. An ultrathin (10 nm) ta-C film was deposited on the top of TNAs by applying a negative bias voltage to the FTO substrate. The ratio of sp^3^ to sp^2^ bonds of the ta-C film was adjusted by the energy of carbon ions. In this work, three kinds of substrate bias voltage were applied to accelerate the carbon ions. As a result, the carbon thin films C1, C2, and C3 are provided with higher, medium, and lower sp^3^-C content, respectively.

### Material Characterizations

The structure of TNAs grown on FTO substrate was characterized by the X-ray diffractometer (XRD, BrukerD8 Discover) with a Cu-Kα 1 radiation (*k* = 1.54 Å) at the scanning speed of 2°/min. The topography of TNAs was observed by field emission scanning electron microscope (SEM, FEI Quanta-400, Holland). The electronic structure of ta-C films was examined by an X-ray photoelectron spectroscopy (XPS, ESCALAB 250, Thermo-VG Scientific). A monochromic Al-Kα source with a spot size of 500 μm and pass energy of 20 eV was used for this measurement. Sample cleaning was not performed before the XPS analysis in order to preserve the elemental and physicochemical state of the sample surface.

### Wettability Measurements

Static contact angle measurements were performed by a video contact angle goniometer (SL2008 Powereach, China) based on the sessile drop method. The mean value was calculated from five individual measurements.

### Cell Culture

Prior to cell culture, the FTO substrates with TNAs were cut into pieces (1.0 × 1.0 cm^2^) and were wrapped by sterilization pack and sterilized by autoclave. After being dried at 60 °C for 24 h, the samples then transferred in individual wells of 24-well culture plates. The HUVECs (ATCC CRL-1730) were cultured in endothelial cell medium (ECM, ScienCell) supplemented with 5 % fetal bovine serum, 1 % endothelial cell growth supplements (ECGS), and 1 % penicillin–streptomycin. Incubation was carried out at 37 °C in an atmosphere of 5 % CO_2_. After 80 % confluence, HUVECs were suspended in complete medium and seeded onto the various substrates at a concentration of 5 × 10^4^ cells per well.

### Cell Viability

After 24 h of culture, the cell viability of HUVECs adhered on different TNA substrates were assessed using a cell-permeable dye calcein-AM (Invitrogen) and a cell-impermeable DNA-binding dye propidium iodide (PI, Sigma-Aldrich). The cells were incubated in phosphate-buffered saline (PBS) containing 5 μg/mL calcein-AM and 50 μg/mL PI at 37 °C for 20 min and then immediately examined under a fluorescence microscope (Leica DM2500). At least eight random regions on each sample were chosen to be photographed, and the mean number of live cells was calculated.

### Cell Attachment and Proliferation

Cell attachment was evaluated by counting the cell numbers on substrates after 4-h incubation. Cell proliferation was determined by measuring the increase in cell numbers from 24 to 72 h of culture without renewal of the medium. At defined time points, the HUVECs cultured on experimental substrates were fixed with 4 % paraformaldehyde in PBS for 20 min at room temperature. Fixed cells were then permeabilized with 1 % Triton X-100 (Sigma-Aldrich) in PBS for 5 min and blocked with 1 % bovine serum albumin (BSA; Sigma-Aldrich) in PBS for 60 min. To examine the cytoskeleton, the F-actin of the fixed cells was incubated with 2 μg/mL phalloidin tetramethyl rhodamine isothiocyanate (TRITC; Sigma-Aldrich) for 60 min. The cells were also counterstained with Hoechst solution (Sigma-Aldrich) to image the nucleus. To determine the cell attachment and proliferation, the mean cell number of each substrate was analyzed from at least 10 fields at ×40 magnifications. Hoechst-stained nucleus was counted by using the “Analyze particles” tool in ImageJ software.

### Statistical Analysis

To ensure reproducibility and obtain better statistics, all assays were repeated in triplicate. All data are expressed as means value ± standard error (SE). The data were subjected to one-way ANOVA to determine the statistical difference. In all cases, a *p* value of <0.05 was considered statistically significant.

## Results and Discussion

### Material Characterization

**Fig. 1:**
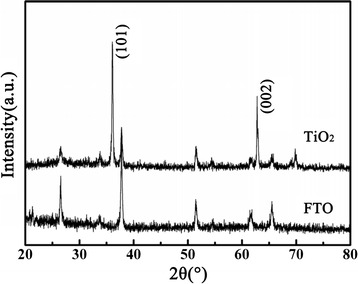
XRD patterns of the FTO substrate before solvent-thermal growth and after

Figure 1 shows the XRD patterns of the FTO substrate before and after solvent-thermal reaction. There are two major diffraction peaks that appear in Fig. 1b. These diffraction peaks were in good agreement with the tetragonal rutile phase (SG, P42/*mnm*; JCPDS No. 21–1276, *a* = *b* = 0.4593 nm and *c* = 0.2959 nm), and we confirmed that the films deposited on FTO substrates are rutile TiO_2_. Compared to the powder diffraction pattern, the (002) diffraction peak was significantly enhanced and some diffraction peaks including (111), (211), and (220) were absent, which indicates that the TiO_2_ nanorods were well crystallized and grew in the direction preferentially.

**Fig. 2:**
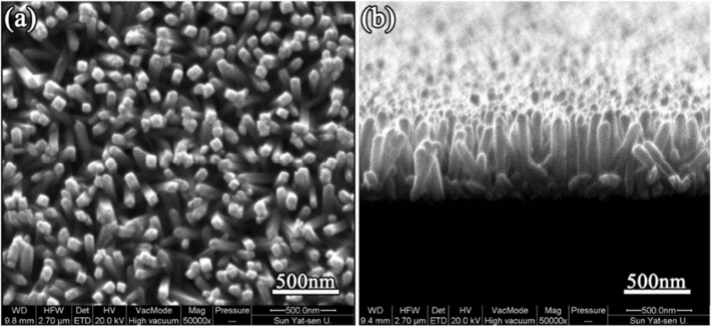
High-magnification (×50,000) SEM images of rutile TiO_2_ nanorod film grown on FTO substrate. **a** Top view. **b** Cross-sectional view. The *scale bar* is 500 nm

Figure 2 shows the typical SEM images of as-synthesized TNA sample. Top and cross-sectional view images show the FTO substrate was covered with nanorods very uniformly, and they grew nearly perpendicular to the FTO substrate. The areal density of TNAs was 40 nanorods/μm^2^. The average length of nanorods was 500 nm, and the average diameter was 100 nm.

**Fig. 3:**
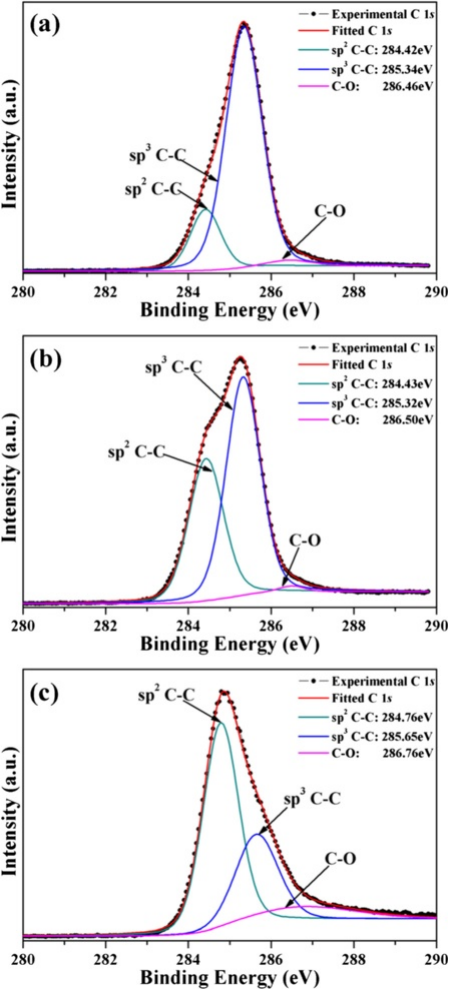
C 1*s* XPS spectra and its fitting of ta-C films deposited at different bias. **a** −100 V, **b** −300 V, and **c** −900 V

Figure 3 shows the C 1*s* XPS spectra of ta-C films deposited at bias voltages of −100, −300, and −900 V, respectively. After employing a Shirley inelastic background subtraction [20, 21], the C 1*s* spectra of each sample was fitted with three Gaussian distributions which are assignable to sp^2^ hybridized carbon, sp^3^ hybridized carbon (shifted from the sp^2^ peak by ~1 eV), and C–O bond. The relative content of sp^3^ hybridization was estimated from the integrated area ratio of sp^3^ peak over the total C 1*s* peak [21]. The relative content of sp^2^-C, sp^3^-C, and C–O components was stated in Table 1. Results show the deposited bias has a substantial effect on the surface sp^2^/sp^3^ ratio. The sp^3^-C contents in samples C1, C2, and C3 were 83.2, 59.4, and 30.4 %, respectively, which are coincidence with our previous research by Raman spectroscopy analysis [18].

**Table 1:** Relative fraction of sp^2^, sp^3^, and C–O components for ta-C films deposited at different biases

Bias	Bonds		
	sp^2^ C–C (%)	sp^3^ C–C (%)	C–O (%)
C1 (−100 V)	15.6	83.2	1.2
C2 (−300 V)	38.3	59.4	2.3
C3 (−900 V)	58.2	30.4	11.4

**Fig. 4:**
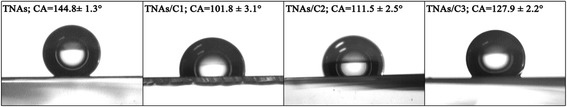
Typical optical micrograph of deionized water droplets on the pure TNAs and different ta-C film-coated TNAs. Contact angles are indicated

Figure 4 shows the water contact angle (CA) of TNAs and TNAs/ta-C. The pure TNAs exhibited an approximate superhydrophobic surface with a static CA of 144.8 ± 2.6° whereas the TNAs/ta-C exhibited relatively low levels. In this work, the carbon films we deposited were about 10-nm thickness, which was unable to transform the surface geometric topography of TNAs. Thus, the transition of wettability between the TNAs and TNAs/ta-C attributed to the alteration of surface chemical composition. Additionally, the sp^2^-rich surfaces present higher contact angle than sp^3^-rich surfaces [22]. It was reported that water CA for natural diamond single crystals (111) and graphite (001) is 35° and 78°, respectively [23]. According to deposition mechanism, the surface atoms of ta-C have dangling bonds [24]. For minimizing the surface energy, the surface reconstructed to remove some of these dangling bonds, and this is usually done by the formation of sp^2^ sites. This was confirmed by theoretical works [25] and experimental results [26].

### Cell Attachment

**Fig. 5:**
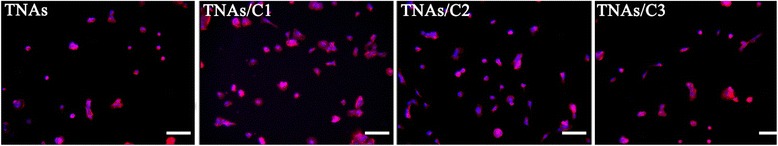
Typical fluorescence images of HUVECs cultured for 4 h on the respective surfaces. The s*cale bar* is 100 μm

**Fig. 6:**
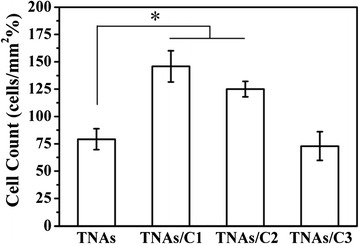
The cell density of HUVECs attached onto surfaces after 4-h culture

Cell attachment was evaluated by analyzing the cell numbers on substrates after 4-h incubation. Figure 5 showed the typical fluorescence imaging of cell attachment after 4-h incubation, and the statistical results were shown in Fig. 6. Cell densities on surfaces of TNAs/C1 and C2 were significantly greater than that of pure TNAs, and there were no major differences between the TNAs and TNAs/C3 (*p* > 0.05). Furthermore, the cell number per unit area increased as the CA decreased, denoting that cell attachment was favored on more hydrophilic surfaces. Surface wettability is proven to be an important factor that has effect on cell attachment. Chai et al. noted in particular that wettability generally acts more directly on initial cell adhesion behavior (within 2 h in culture) [27]. Ma et al. consider that higher surface energy generally results in better cell adhesion in the beginning of cells seeding on material surface [28]. Zhao et al. found that quasi-aligned nanowire arrays (titanium carbide-carbon) with a high water CA (137.5°) could repel cell adhesion [29], but they regarded nanostructure as the primary factor irrespective of surface chemistry and wettability.

### Cell Viability

**Fig. 7:**
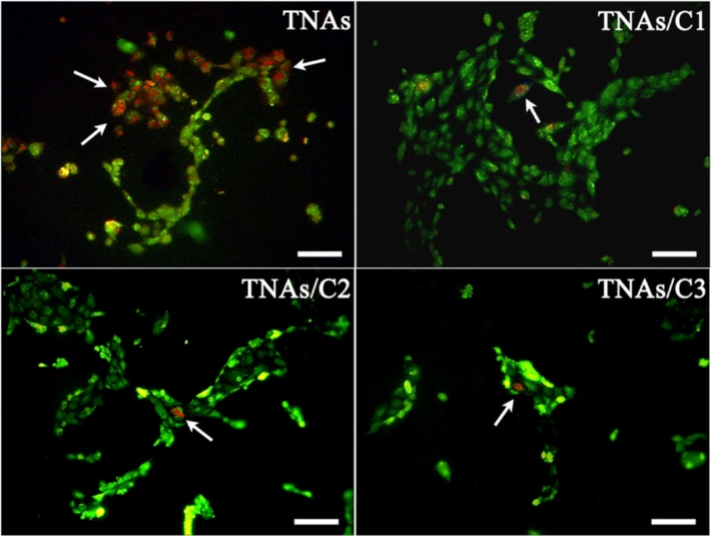
The typical fluorescence microscopy images of live (*green*) and dead (*red*) cells on various surfaces. The *scale bar* is 100 μm

**Fig. 8:**
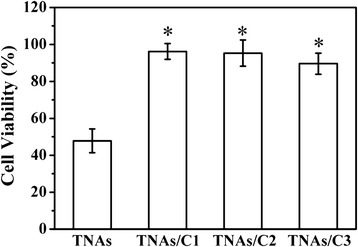
Quantification of cell viability for various substrates (*n* = 3). The *asterisk* denotes that the carbon-coated TNA is significantly higher than pure TNAs (*p* < 0.05)

HUVEC viability was quantitatively measured with PI/calcein-AM double staining after 24 h of the incubation. Figure 7 showed the typical fluorescence imaging of cell viability assays. Stained live cells (green) and dead cells (red) can be easily identified by fluorescence microscopy. The yellow cells were overlap of live and dead cells. The statistical results (Fig. 8) revealed that cells growth on both TNAs/C1 and TNAs/C2 kept a good viability over the time of cell culture and more than 97 % of cells were alive. The cells cultured on TNAs/C3 also represented a relativity high viability. However, the cells cultured on bare TNAs exhibited a poor viability (48 %). It is interesting to contrast our results with the work of Kim et al. [30]. They found the mammalian cells could survive on silicon nanowire arrays for several days, in spite of the cells were penetrated by the vertical nanowires. In their work, only 20–30 nanowires were exposed to each cell, it seemed that such low-density was hardly causing the enough toxicity to the cells, and therefore the cells survived.

In our experiment, ~15,000–20,000 TiO_2_ nanorods were exposed to each cell. Thus, we raised the possibility that a large number of TNAs were engulfed by cells. If that is the case, the long-term toxicity caused by TNA engulfment may lead to the cell death. Indeed, the TiO_2_-based nanomaterials (including nanotubes, nanowires, and nanoparticles) have been widely reported to be cytotoxic in mammalian cells, inducing cell death by apoptosis and necrosis [31]. The results of Lee et al. suggested that ZnO nanorods with diameter similar to ours are engulfed by umbilical vein endothelial cells also lead the cells death [32]. But they considered that the cell adhesion and survival has no obvious relationship with the surface chemistry of ZnO nanorods (with or without silicon dioxide coating) [33]. However, the surface chemical properties play an important role in our work. The a-C film-coated TNAs exhibited superior cell viability compare to the pure TNAs. We considered it was mainly attributed to the ultrathin carbon coating, which acted as a barrier preventing the delivery of toxic material into cells.

### Cell Proliferation

**Fig. 9:**
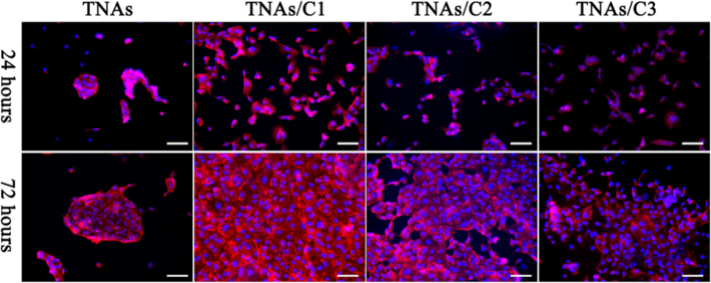
Typical fluorescent microscopy images of HUVECs cultured for 24 and 72 h on the respective substrates. The *scale bar* is 100 μm

**Fig. 10:**
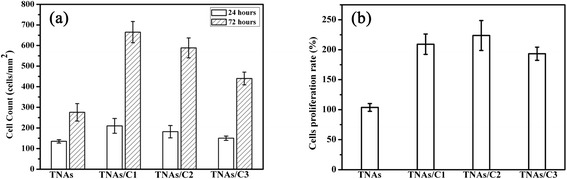
**a** The cell density of HUVECs cultured on different substrates over a period of 72 h. **b** Proliferation rates of HUVECs were represented by relative increase in cell number from 24- to 72-h incubation

Cell proliferation rate was investigated by analyzing the relative increase in cell number from 24 to 72 h in culture. Figure 9 shows the typical growth of HUVECs after culturing at defined time points. Notably, the cells cultured on TNAs/C1 became fully confluent after 72 h culturing, but they exhibit a poor confluent on pure TNAs and most of them were single or tend to clustering. The statistical result (Fig. 10a) shows that the cell density on TNAs/C1 reached to (665 ± 52) cells/mm^2^, whereas in case of TNAs, this value was 275 ± 42 cells/mm^2^. The cell densities of TNAs/C2 and TNAs/C2 were 588 ± 47 and 440 ± 31 cells/mm^2^, respectively. After 72 h, TNAs/C1 and TNAs/C2 induced a cell proliferation rate of more than 200.0 % (Fig. 10b). The TNAs/C3 also induced a high rate of cell proliferation (193.3 %), whereas the pure TNAs produced the lowest proliferation rates of 103.7 %.

These phenomena could be attributed to the changes in surface wettability and chemical composition after carbon plasma coating. Surface wettability is believed to be an important factor that guides the first events occurring at the cell/biomaterial interface, such as interaction of medium and proteins with biomaterial and subsequent cell responses [34]. The superhydrophobic TNA substrate not only results in poor cell attachment in the initial seeding (Figs. 7 and 8) but also cause the further inhibition of cell proliferation and cell clustering because the weak cell-surface interaction [35]. In addition, we cannot rule out the possibility that long-term toxicity of TiO_2_ nanorods due to engulfment, which would decrease cell survival and suppress cell proliferation subsequently at the long times. More detailed studies are needed to investigate this possibility.

On the other hand, the TNAs after carbon plasma treating exhibited a superior proliferative capacity. Our data suggested that the ta-C films play an important role in regulating cell proliferation, which not only shielded the HUVECs from toxicity but also regulated the wettability by electronic structure (sp^2^/sp^3^ rate). The a-C film-coated TNAs with the highest sp^3^-C content result in lowest water CA (101.8°) and present the best cell attachment and proliferation, whereas the pure TNAs (CA = 144.8°) do the opposite thing. Our results are consistent with the work of Ranella et al. [12]. They suggested that cell response shows a non-monotonous and sigmoidal dependence on the synergy of surface roughness and chemistry, which determines the wettability or surface energy of the culture substrate (3D micro/nanosilicon surfaces). They revealed that optimal cell adhesion and spreading was obtained on the substrate of small roughness and moderate wettability (CA = 105°). On the contrary, they founded cell response was effectively inhibited on highly rough and superhydrophobic substrates (CA = 152°).

### Cell Morphology

**Fig. 11:**
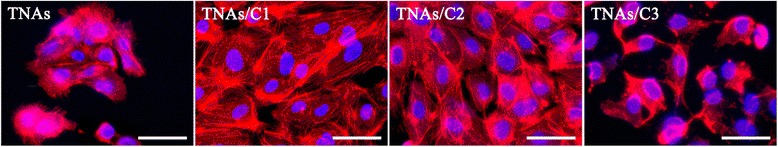
The typical fluorescent microscopy images of cell morphology after 48 h. The *scale bar* is 50 μm

The fluorescent microscopy images showed the morphological changes of HUVECs cultured on the various substrates here (Fig. 11). The majority of HUVECs cultured on both TNAs/C1 and C2 showed a normal, cobblestone-like shape that resembled the morphology of HUVECs in vivo. These cells exhibited well-developed cytoskeleton, which spans over the cell body, and the actin fibers appeared with a longitudinal organization and stress state. Furthermore, the high-density organization of actin filament bundles was found at the junction of cells. Conversely, the cells cultured on pure TNAs and TNAs/C3 exhibited an abnormal morphology. The cell body appeared much smaller, in ordinance and un-spread. And the weak and vague fluorescent structures with less actin fibers were detected. Unusual cell shape and lack of cell spreading can cause cell death in each of the cell types studied here [36], which may explain the observed decrease in cell survival and proliferation rate of TNAs. Similar results were also obtained by culturing HUVECs on high-density Al_2_O_3_ nanowires [37] and ZnO nanorods [31]. Cell processes such as proliferation and apoptosis are arbitrated by cell shape and cytoskeletal organization directly determined by cell/surface interaction [38]. The interaction of cells with a given material surface is dependent upon both surface topography and chemistry.

## Conclusions

In this work, the ta-C films with controlled fraction of sp^3^-C were deposited on TNAs without changing the surface topography of substrates. The wettability of substrates was determined by sp^3^ to sp^2^ ratio, and the sp^3^-C-rich surfaces present more hydrophilic than sp^2^C-rich surfaces. The adhesion, viability, proliferation, and morphology of HUVEC cells cultured on TNAs and TNAs/ta-C have been investigated. It was found that the carbon nanocoatings significantly improved cell viability. In addition, the cells were likely to attach on high sp^3^-C-contented surfaces and exhibit better shape configuration and proliferation. Our data indicate that ta-C film-coated TNAs possess superior cytocompatibility. The excellent cell compatibility is mainly ascribed to the nontoxic properties and moderate wettability of ta-C films adjusted by sp^3^ to sp^2^ ratio.

## References

[1] Li G, Ping Y, Wei Q, Maitz MF, Zhou S, Nan H (2011). The effect of coimmobilizing heparin and fibronectin on titanium on hemocompatibility and endothelialization. Biomaterials.

[2] Lin Q, Yan J, Qiu F, Song X, Fu G, Jian J (2011). Heparin/collagen multilayer as a thromboresistant and endothelial favorable coating for intravascular stent. Journal of Biomedical Materials Research Part A.

[3] Rogers C, Parikh S, Seifert P, Edelman ER (1996). Endogenous cell seeding. Remnant endothelium after stenting enhances vascular repair. Circulation.

[4] Kushwaha M, Anderson JM, Bosworth CA, Andukuri A, Minor WP, Lancaster JR (2009). A nitric oxide releasing, self assembled peptide amphiphile matrix that mimics native endothelium for coating implantable cardiovascular devices. Biomaterials. Biomaterials..

[5] Lu Q, Zhang S, Hu K, Feng Q, Cao C, Cui F (2007). Cytocompatibility and blood compatibility of multifunctional fibroin/collagen/heparin scaffolds. Biomaterials.

[6] Gong F, Cheng X, Wang S, Zhao Y, Yun G, Cai H (2010). Heparin-immobilized polymers as non-inflammatory and non-thrombogenic coating materials for arsenic trioxide eluting stents. Acta Biomaterialia.

[7] Meng S, Liu Z, Shen L, Guo Z, Chou LL, Zhong W (2009). The effect of a layer-by-layer chitosan–heparin coating on the endothelialization and coagulation properties of a coronary stent system. Biomaterials.

[8] Milner KR, Snyder AJ, Siedlecki CA (2006). Sub-micron texturing for reducing platelet adhesion to polyurethane biomaterials. Journal of Biomedical Materials Research Part A.

[9] Sun T, Tan H, Han D, Fu Q, Jiang L (2005). No platelet can adhere—largely improved blood compatibility on nanostructured superhydrophobic surfaces. Small.

[10] Yun Y, Lai Y, Zhang Q, Ke W, Zhang L, Lin C (2010). A novel electrochemical strategy for improving blood compatibility of titanium-based biomaterials. Colloids & Surfaces B Biointerfaces.

[11] Kim SI, Jin IL, Bo RL, Mun CH, Jung Y, Kim SH (2014). Preparation of lotus-leaf-like structured blood compatible poly(ɛ-caprolactone)-block-poly(l-lactic acid) copolymer film surfaces. Colloids & Surfaces B Biointerfaces..

[12] Ranella A, Barberoglou M, Bakogianni S, Fotakis C, Stratakis E (2010). Tuning cell adhesion by controlling the roughness and wettability of 3D micro/nano silicon structures. Acta Biomaterialia.

[13] Bacakova L, Filova E, Parizek M, Ruml T, Svorcik V (2011). Modulation of cell adhesion, proliferation and differentiation on materials designed for body implants. Biotechnol Adv.

[14] Ding Y, Yang Z, Bi CWC, Yang M, Xu SL, Lu X (2014). Directing vascular cell selectivity and hemocompatibility on patterned platforms featuring variable topographic geometry and size. ACS Applied Materials & Interfaces.

[15] Brammer KS, Choi C, Frandsen CJ, Oh S, Johnston G, Jin S (2011). Comparative cell behavior on carbon-coated TiO_2_ nanotube surfaces for osteoblasts vs. osteo-progenitor cells. Acta Biomaterialia.

[16] Rodil SE, Olivares R, Arzate H, Muhl S (2003). Properties of carbon films and their biocompatibility using in-vitro tests. Diamond & Related Materials.

[17] Luo P, Huang ZY, Chen DH (2011). Preparation and the blood compatibility of titanium oxide nanorod arrays. Advanced Materials Research..

[18] Chen HP, Chen HL, Chen DH, Chen M (2014). Synergistic effect of carbon microstructure and topography of TiO_2_ nanorod arrays on hemocompatibility of carbon/TiO_2_ nanorod arrays composites. Journal of Materials Science.

[19] Chen HL, Luo P, Huang ZY, Chen HP, Chen M, Chen DH (2013). Preparation and blood compatibility of carbon/TiO2 nanocomposite. Diamond & Related Materials.

[20] Shirley DA (1972). High-resolution X-ray photoemission spectrum of the valence bands of gold. Phys Rev B.

[21] Niakan H, Yang Q, Szpunar JA (2013). Structure and properties of diamond-like carbon thin films synthesized by biased target ion beam deposition. Surface & Coatings Technology.

[22] Ostrovskaya LY, Dementiev AP, Kulakova II, Ralchenko VG (2005). Chemical state and wettability of ion-irradiated diamond surfaces. Diamond & Related Materials.

[23] Piazza F, Morell G (2009). Wettability of hydrogenated tetrahedral amorphous carbon. Diamond & Related Materials.

[24] Robertson J (2002). Diamond-like amorphous carbon. Materials Science & Engineering R Reports.

[25] Haerle R, Galli G, Baldereschi A (1999). Structural models of amorphous carbon surfaces. Appl Phys Lett.

[26] Libassi A, Ferrari AC, Stolojan V, Tanner BK, Robertson J, Brown LM (2000). Density, sp^3^ content and internal layering of DLC films by X-ray reflectivity and electron energy loss spectroscopy. Diamond & Related Materials.

[27] Feng C, Mathis N, Blanchemain N, Meunier C, Hildebrand HF (2008). Osteoblast interaction with DLC-coated Si substrates. Acta Biomaterialia.

[28] Martin PJ, Bendavid A, Liu Z, Ionescu M, Zreiqat H (2007). DLC coatings: effects of physical and chemical properties on biological response. Biomaterials.

[29] Zhao L, Hu L, Huo K, Zhang Y, Wu Z, Chu PK (2010). Mechanism of cell repellence on quasi-aligned nanowire arrays on Ti alloy. Biomaterials.

[30] Kim W, Ng JK, Kunitake ME, Conklin BR, Yang P (2007). Interfacing silicon nanowires with mammalian cells. Jamchemsoc.

[31] Magrez A, Horváth L, Smajda R, Salicio V, Pasquier N, Forró L (2009). Cellular toxicity of TiO_2_-based nanofilaments. Acs Nano.

[32] Jiyeon L, Kang BS, Barrett H, Chancellor TF, Byung Hwan C, Hung-Ta W (2008). The control of cell adhesion and viability by zinc oxide nanorods. Biomaterials.

[33] Jiyeon L, Byung Hwan C, Ke-Hung C, Fan R, Lele TP (2009). Randomly oriented, upright SiO_2_ coated nanorods for reduced adhesion of mammalian cells. Biomaterials.

[34] Tzoneva R, Faucheux N, Groth T (2007). Wettability of substrata controls cell-substrate and cell-cell adhesions. Biochimica Et Biophysica Acta.

[35] Jung Yul L, Shaughnessy MC, Zhiyi Z, Hyeran N, Vogler EA, Donahue HJ (2008). Surface energy effects on osteoblast spatial growth and mineralization. Biomaterials.

[36] Chen CS, Mrksich M, Huang S, Whitesides GM, Ingber DE (1997). Geometric control of cell life and death. Science.

[37] Aktas C, Dörrschuck E, Schuh C, Miró MM, Lee J, Pütz N (2012). Micro- and nanostructured Al_2_O_3_ surfaces for controlled vascular endothelial and smooth muscle cell adhesion and proliferation. Materials Science & Engineering C.

[38] Lipski AM, Pino CJ, Haselton FR, Chen IW, Shastri VP (2008). The effect of silica nanoparticle-modified surfaces on cell morphology, cytoskeletal organization and function. Biomaterials.

